# Identification and expression of troponin T, a new marker on the surface of cultured tumor endothelial cells by aptamer ligand

**DOI:** 10.1002/cam4.260

**Published:** 2014-05-09

**Authors:** Mst Naznin Ara, Mamoru Hyodo, Noritaka Ohga, Kosuke Akiyama, Kyoko Hida, Yasuhiro Hida, Nobuo Shinohara, Hideyoshi Harashima

**Affiliations:** 1 Laboratory of Innovative Nanomedicine, Faculty of Pharmaceutical Sciences, Hokkaido UniversityKita 12, Nishi 6, Kita-ku, Sapporo, Hokkaido, 060-0812, Japan; 2 Division of Vascular Biology, Graduate School of Dental Medicine, Hokkaido UniversityKita 13, Nishi 7, Kita-ku, Sapporo, Hokkaido, 060-0812, Japan; 3 Department of Cardiovascular and Thoracic Surgery, Graduate School of Medicine, Hokkaido UniversityKita 15, Nishi 7, Kita-ku, Sapporo, Hokkaido, 060-0812, Japan; 4 Department of Urology, Graduate School of Medicine, Hokkaido UniversityKita 15, Nishi 7, Kita-ku, Sapporo, Hokkaido, 060-0812, Japan

**Keywords:** Biomarker, DNA aptamer, MALDI-TOF-mass spectroscopy, mTECs, troponin T

## Abstract

The identification of a specific biomarker involves the development of new clinical diagnostic tools, and an in-depth understanding of the disease at the molecular level. When new blood vessels form in tumor cells, endothelial cell production is induced, a process that plays a key role in disease progression and metastasis to distinct organs for solid tumor types. The present study reports on the identification of a new biomarker on primary cultured mouse tumor endothelial cells (mTECs) using our recently developed high-affinity DNA aptamer AraHH001 (*K*_d_ = 43 nmol/L) assisted proteomics approach. We applied a strategy involving aptamer-facilitated biomarker discovery. Biotin-tagged AraHH001 was incubated with lysates of mTECs and the aptamer-proteins were then conjugated with streptavidin magnetic beads. Finally, the bound proteins were separated by sodiumdodecyl sulfate polyacrylamide gel electrophoresis (SDS-PAGE) with silver staining. We identified troponin T via matrix assisted laser desorption ionization-time of flight (MALDI-TOF) mass spectrometry, the molecular target of aptamer AraHH001, and its presence was confirmed by measuring mRNA, protein levels, western blot, immunostaining, a gel shift assay of AraHH001 with troponin T. We first report here on the discovery of troponin T on mTECs, a promising and interesting diagnostic tool in the development of antiangiogenic therapy techniques the involves the targeting of the tumor vasculature.

## Introduction

Biomarkers are generally recognized as indicators of a specific biological state and play an important role in all living organisms as well as in various pathological conditions. It has been estimated that a cancer proteome may include more than 1.5 million proteins due to the wide variety of posttranslational processing and modifications that are possible. In comparison, very few have been identified 1,2. Because of the complexity of this situation, the identification of biomarkers that permit oncology problems to be addressed remains an important issue. Generally, tumor-related membrane proteins are involved in cell signaling, cell–cell interactions, alterations in membrane permeability by facilitating ion or solute transport between outer environments to the cells, which contribute to transforming healthy cells into disease cells 3–5.

Cancer is often associated with angiogenesis 6,7. Angiogenesis is defined as a complex endothelial cell growth that proceeds through interaction of different growth factors in association with many markers that are expressed to aid angiogenesis. The key mediator of angiogenesis is VEGF (vascular endothelial growth factor) an angio-protein that opens the possibility of selectively targeting specific pathways for developing antiangiogenesis therapy 8. Cytokines also modulate angiogenesis by regulating VEGF expression 9. The Croix group first reported on specific markers associated with tumor endothelial cells (TECs) using a genomic approach, and designated them as tumor endotheial markers 10. Many other TEC-specific markers have been identified such as CD13, CD31, CD105, CD144, etc. 11. Specific gene expression signatures have been reported in endothelial cells and blood lymphocytes in response to antiangiogenic drugs, such as the VEGFR2 inhibitor SU5416, endistatin, and brivanib alaninate, a VEGFR2/fiblioblast growth factor receptor-1 inhibitor 8. However, available methods or technologies for monitoring the activities of drugs, and their response to patient outcomes are not sufficient. Several molecules from TECs, which are preferentially expressed during the angiogenic process, have been proposed as a target epitope, and numerous strategies have been pursued for achieving this 2,12.

Matrix assisted laser desorption ionization-time of flight (MALDI-TOF) mass spectrometry can be used to identify biomarkers that are expressed specifically or at different levels on different disease specimens, from serum samples of membrane proteins of different tumor tissues, tumor cell lines, and, or even cultured TECs 5,13–15. However, the identification of specific target protein biomarkers related to disease continues to be a challenge and represents an urgent need.

Aptamers are oligonucleotide structured ssDNA or ssRNA 16. Aptamers are generally nontoxic and low molecular weight (8–15 kDa) 17. The screening of aptamers through SELEX (Systemic Evolution of Ligands by EXponential enrichment) from combinatorial libraries by an iterative in vitro selection approach was first independently reported by the Ellington and Tuerk groups in the early 1990s 18,19. Cell-SELEX is a modified version of SELEX that functions against complex live target cells. In the last two decades, aptamers have attracted interest for use as an efficient molecular probe due to their numerous therapeutic applications across a wide range of diverse and complex targets 20 including small molecules such as dyes, metal ions, amino acids and nucleotides, biomolecules such as nucleic acids and proteins, molecular complexes, viruses, whole organisms, or even live cells 21–36.

Aptamer-mediated biomarker discovery is now a very promising approach for the diagnosis of diseases by biomolecular recognition-based applications 37–43. Our objective was to identify a novel biomarker from mouse tumor endothelial cells (mTECs) using our recently developed DNA aptamer AraHH001-assisted proteomics methodology 44.

## Materials and Methods

### Isolation of primary cultured mTECs and normal skin endothelial cells

All experiments involving animals and their care were carried out consistent with Hokkaido University guidelines, and protocols approved by the Institutional Animal Care and Use Committee (experiments using mice were approved by the Pharmaceutical Science Animal Committee of Hokkaido University). Endothelial cells were isolated as previously described 45–48. Briefly, normal skin endothelial cells (skin-ECs) were isolated from the dermis as controls. mTECs and skin-ECs were isolated by magnetic bead cell sorting using an IMag cell separating system (BD Bioscience, San Jose, CA). CD31-positive cells were sorted and plated on 1.5% gelatin-coated culture plates and grown in EGM-2 MV (Clonetics, Walkers, MD) and 15% fetal bovine albumin (FBS). Diphtheria toxin (DT) (500 ng/mL; Calbiochem, San Diego, CA) was added to mTEC subcultures to kill any remaining human tumor cells and to the normal skin-ECs subcultures for technical consistency. Human cells express the heparin-binding EGF-like growth factor (hHB-EGF), a DT-receptor. However, DT does not interact with mouse HB-EGF and murine ECs survive this treatment. The isolated skin-ECs were purified by a second round of purification using FITC-BS1-B4. They were cultured in EGM-2 MV (Lonza, Basel, Switzerland) and 15% FBS.

### Maintenance of cell cultures

mTECs and skin-ECs were cultured using a special media, namely EGM-2 MV (Lonza). To prevent microbial growth, penicillin (100 U/mL) and streptomycin (100 *μ*g/mL) were added to EGM-2 MV. Cell cultures were maintained at 37°C in a 5% CO_2_ incubator at 95% humidity. For regular cell cultures a 0.1% trypsin solution was used to dissociate the cells from the surface of the culture dish. However, during the entire selection of a DNA aptamer, a flow cytometry assay and during aptamer-targeted protein purification, RepCell 44 dish was used (CellSeed Inc. Tokyo, Japan).

### Fluorescence activated cell sorting analysis

**Figure 1:**
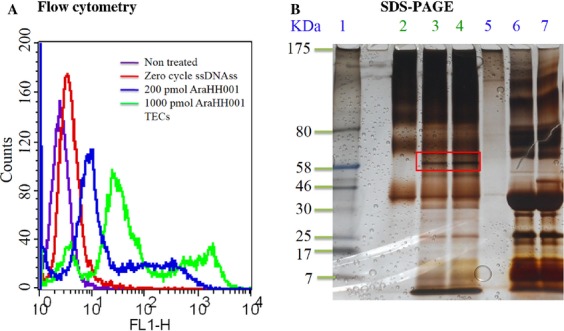
A new DNA aptamer AraHH001 binds to mTECs, and isolated target protein troponin T via aptamer protein pulls down from mTECs. (A) Flow cytometry-binding assay of the selected FITC-tagged AraHH001 DNA aptamer with mTECs. The purple, red, blue, and green curve represents untreated cells, treated cells with a 200 pmol zero cycle ssDNAs pool, 200 pmol and 1000 pmol FITC-tagged AraHH001, respectively. (B) SDS-PAGE analysis of the aptamer AraHH001-based purification of the target protein. Lane 1 represents the protein marker (7–175 kDa), lane 2 represents proteins treated with magnetic beads (MB) with a whole cell lysates and lanes 3 and 4 represents aptamer-bound target proteins treated with MB without and with a linker. Lanes 5, 6, and 7 represents unbound fractions from a whole cell lysates, aptamer without linker and aptamer with linker, respectively.

To evaluate the binding affinity of selected aptamer AraHH001 (Fig.1A), it was heated in 500 *μ*L of 1× selection buffer (50 mmol/L Tris-HCl, 5 mmol/L KCl, 100 mmol/L NaCl, 1 mmol/L MgCl_2_, 250 mmol/L sucrose, and 0.1% sodium azide) at 80°C for 10 min and then slowly cooled to permit the formation of secondary structures. We added a 5 mol/L excess of yeast tRNA and BSA as a blocking agent. It should be noted here that we prepared cells using RepCell dish to avoid damage to the cell surface proteins caused by the use of trypsin and we also filtered the cells using a 40 *μ*mol/L Cell Strainer (BD Falcon, Franklin lakes, NJ) to remove clumped cells. We then incubated the 1 × 10^6^ cells on ice for 45 min with the aptamer and, or with zero cycle ssDNA libraries as a negative control. The cells were spun down (5000*g*, 5 min at 4°C) to remove the supernatant that contains unbound DNA. The cells were washed two to three times with 1× selection buffer. Final analysis was performed by flow cytometry (BD Biosciences) by counting 10,000 events.

A fluorescence activated cell sorting (FACS) experiment was performed to check the expression of the identified troponin T protein on mTECs using a FITC-conjugated cardiac troponin T (cTnT) antibody. A total of 1 × 10^6^ mTECs was collected for a nontreated and or a treated with excess 8 *μ*L FITC-conjugated cTnT antibody (0.9 mg/mL) in 500 *μ*L of 1× selection buffer and then incubated on ice for 50 min. The cells were washed twice with 1× selection buffer and the fluorescence detected by measuring 10,000 counts using flow cytometry 49. To confirm membrane protein binding, excess FITC-tagged anti-troponin T antibody was incubated with 0.1× trypsin pretreated mTECs for 50 min, washed twice and analyzed by 10,000 counts using flowcytometry.

### Aptamer-targeted protein purification and peptide mass fingerprinting analysis

During the experiment, mTECs (6 × 10^6^ cells) were collected and washed three times with 1× PBS followed by filtration through a 40-*μ*m cell strainer (BD Falcon) to remove clumped cells. The cells were then lysed in lysis buffer (1% triton X-100, 5 mmol/L MgCl_2_, 20 mmol/L phenylmethylsulfonyl fluoride [PMSF], 0.1% protease inhibitor) at 4°C on ice for 30 min. After centrifugation (14,000*g*, 5 min, 4°C), cell debris was removed and the proteins containing in the supernatant was collected 31. We prepared three equal volumes of protein solutions. Only cellular proteins were used as a control. The other two samples were incubated with DNA aptamer AraHH001 with or without linkers (Fig. S1). In both cases, we used 2 nmol of biotin-tagged aptamer. As a linker, we used 5 nmol DTSSP. We examined both conditions, because sometimes an aptamer only is not sufficient to permit the target protein to be identified. To conjugate the aptamer with the linker, we incubated the aptamer and linker for 2 h at 4°C followed by incubation with cellular protein lysates for 1 h on ice. Each of the samples was next mixed with streptavidin-coated magnetic beads 2 mL (Promega, Fitchberg, WI), followed by incubation for 10 min at room temperature. After the incubation, the magnetic beads were washed three times and finally the target-bound protein was eluted from the magnetic beads by heating at 95°C for 5 min in 50 *μ*L of the elution buffer, which was then used in the sodiumdodecyl sulfate polyacrylamide gel electrophoresis (SDS-PAGE) analysis with silver staining (Fig.1B). To identify the desired protein, the band was excised and subjected to a peptide mass fingerprinting (PMF) analysis by Genome Inc. 2,3 (Table S1, Fig. S2A and B).

### Quantitative real-time RT-PCR

Total RNA was isolated from mTECs 47,50, as previously described. Briefly, total RNA was extracted using an RNeasy Micro Kit (Qiagen, Valencia, CA) using the RNase-free DNase set (Qiagen). RNA was quantified by spectrophotometry. RNA was used for first-strand complementary DNA (cDNA) synthesis in Rever Tra-Plus (Toyobo, Osaka, Japan). The cDNA was amplified by the polymerase chain reaction (PCR). Quantitative real-time RT-PCR (qPCR) was performed using SsoFast™ EvaGreen® Supermix (Bio-Rad, Hercules, CA). Cycling conditions followed the manufacturer's instructions based on the use of CFX Manager (Bio-Rad). The relative expression levels of TNNTI mRNA in TECs and Skin-ECs (NEC, Tokyo, Japan) were normalized to glyceraldehyde 3-phosphate dehydrogenase (GAPDH). The primers are as follows: mouse GAPDH (mGAPDH): *forward*: 5′-TCTGACGTGCCGCCTGGAG-3′; *reverse*: 5′-TCGCAGGAGACAACCTGGTC-3′ mouse troponin T1, Skeletal, Slow (TNNT1): *forward*: 5′-TTGGAGGCCGCTGGAAGTGAGA-3′; *reverse*: 5′-ACAGATGGGACACGCTCCAGTG-3′.

### Electrophoretic mobility shift assay

In the electrophoretic mobility shift assay (EMSA) 51 the Cy5 tagged AraHH001 aptamer in 1× selection buffer was heated in 80°C for 10 min to permit secondary structures to form, and 10 nmol/L of Cy5 tagged AraHH001 was then incubated with a different concentration of cTnT on ice in 4°C for 30 min. Finally, all of the reaction mixture was run on 8% optimized native PAGE. AraHH001 showed a substantial shift and was detected by means of a Luminescent image analyzer (Image Quant LAS-4000; GE Healthcare, Little Chalfont, U.K.).

### Western blot analysis

In western blot (WB) experiment, the cell lysate containing protein samples obtained from mTECS were run on SDS-PAGE and the proteins separated. We ran the cTnT protein as a positive control and prestained protein marker as a ladder. All the samples were electro-transferred from the gel to the PVDF membrane (GE Healthcare) at 70 V for 2 h. The nitrocellulose membrane was activated with 100% methanol for 1 min and rinsed with 1× TBST (Tris buffered saline, 0.1% Tween) buffer before preparing the stack. The membrane was blocked with ECL prime WB detection reagent (GE Healthcare) overnight at 4°C. The membrane was washed three times with 1× TBST buffer at room temperature in shaker. Each washing time was 10–15 min. Then the membrane was stained with primary anti-troponin T monoclonal antibody (Thermo Scientific, Waltham, MA) in a dilution 1:1000 for 2 h at room temperatures in shaker. The membrane was washed three times with 1× TBST buffer three times at room temperatures in a shaker, and stained with secondary a horseradish peroxidase-conjugated goat anti-mouse IgG antibody (Biolegend, San Diego, CA) in a dilution 1:2000 for 2 h at room temperatures in a shaker. The membrane was subjected to washing same as primary antibody treatment. Finally, the membrane was soaked for 5 min with ECL prime detection reagent (GE Healthcare) and a picture obtained by means of a Luminescent image analyzer (Image Quant LAS-4000; GE Healthcare).

### Immunostaining

Human tissue samples were obtained from excised renal cell carcinoma tissues of three patients at the Hokkaido University Hospital. Frozen sections of excised tissue were prepared as described previously 52. Human sections were double stained with Alexa Fluor 647-anti human CD31antibody and FITC-conjugated mouse anti-cTnT antibody (HyTest, Turku, Finland) to assess troponinT colocalization in TECs. All samples were examined using an Olympus FluoView FV10i confocal laser scanning microscope (Olympus, Tokyo, Japan).

## Results and Discussion

### The DNA aptamer AraHH001 molecular probes for the target protein identification

We developed a DNA aptamer AraHH001 the targets mTECs using a cell-SELEX method 44. The specificity and affinity of the DNA aptamer AraHH001 was measured by flow cytometry (Fig.1A). We applied two very simple modifications of an aptamer AraHH001 probe in which the 3′ ends were tagged with biotin by conjugation with streptavidin beads, a process that was necessary for eluting the target protein from the bead-biotin aptamer-protein complex, and 5′ ends with an NH_2_ group for attaching a linker DTSSP. The purpose of adding linkers in the 5′ ends of the aptamer was to enhance the ability of the aptamer to recognize the target; the objective was to determine which conditions are best for purifying the aptamer target. In the present study, we observed that the aptamer without a linker showed the same capacity to pick up the target as the aptamer with a linker, indicating that very minimal modification is needed for the successful purification of the target by an affinity pull down assay (Fig. S1).

### Analysis of purified AraHH001-targeted protein by SDS-PAGE and MALDI-TOF for identification

For the analysis of the purified aptamer-targeted protein, we ran the eluted protein samples on SDS-PAGE, followed by silver staining. The SDS-PAGE results were the same with and without a linker aptamer (Fig.1B). The protein bands in lanes 3 and 4, corresponding to the bound the aptamer, were analyzed and compared using the protein marker in lane 1 as well as a control sample without the aptamer in lane 2. From the gel pictures, the band at around a 60 kDa appeared to be highly promising. Therefore, the excised gel band was analyzed by MALDI-TOF to identify the aptamer-targeted protein 14. Interestingly, the PMF analysis 1,3,4,14 results showed that an AraHH001-targeted protein was the mouse slow skeletal muscle troponin T (TNNT1), and showed a maximum similarity with cTnT (Figs. S2 and S3). Biologically, troponin is a complex of three units of troponin I, C, and T along with tropo-myosin and is located on actin filaments, which are essential for the calcium-mediated regulation of skeletal and cardiac muscle contraction 53. The molecular weight of the identified troponin T was 33 kDa, and it appears to exist as a dimer in gel pictures 53.

### Confirmation of the aptamer AraHH001 target protein troponin T

**Figure 2:**
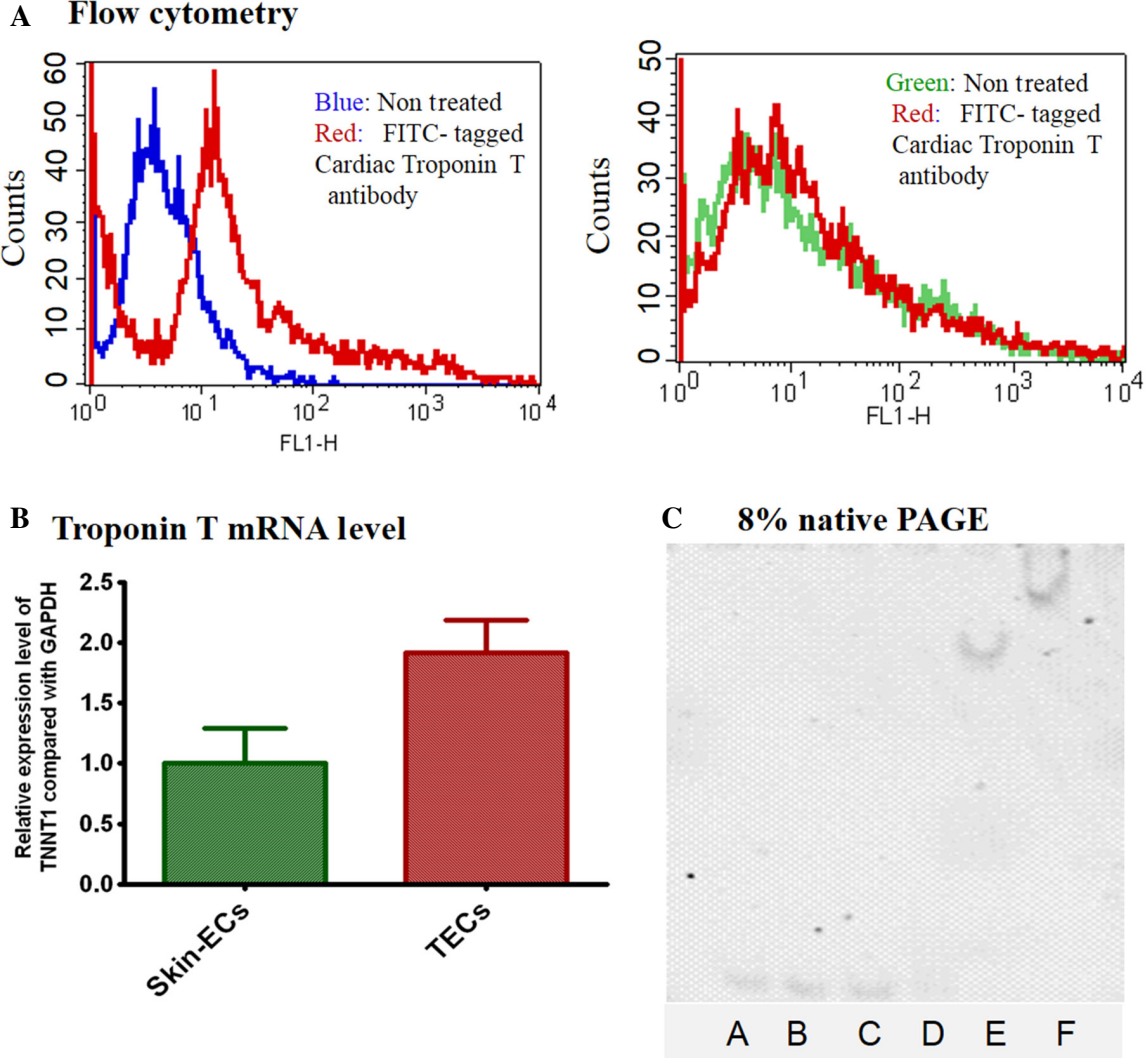
Confirmation of the aptamer AraHH001 target protein troponin T. (A) Troponin T expressed on mTECs. Flow cytometry assay of a FITC-conjugated cardiac troponin T (cTnT) antibody with mTECs and skin-ECs. The blue and red curves represent nontreated and treated mTECs with a FITC-conjugated cTnT antibody. The green and red curves represent nontreated and treated skin-ECs with the FITC-conjugated cTnT antibody. (B) Relative expression of troponin T mRNA level in mTECs and skin-ECs. RT-PCR analyses of the relative expression of troponin T in mTECs as well as in skin-ECs, compared with standard GAPDH. (C) Electrophoretic mobility shift assay (EMSA) of an AraHH001 aptamer with its target protein troponin T. EMSA result represents the binding of 10 nmol/L AraHH001 with 0, 5.33, 16, 80, 400, and 2000 nmol/L cTnT.

We performed a series of experiments to confirm that the target protein was in fact, troponin T. We performed a flow cytometry assay using a FITC-conjugated anti-troponin T cardiac antibody with mTECs and skin-ECs. Surprisingly, the flow cytometry results indicated that troponin T was expressed on mTECs but not on skin-ECs (Fig.2A). The selective expression of troponin T was detected by a troponin T antibody on mTECs and constitutes direct evidence for the selective binding of an AraHH001 aptamer to mTECs. Using qPCR, we further confirmed a higher expression of troponin T levels on mTECs than on skin-ECs by measuring mRNA levels normalized to GADPH (Fig.2B). The above two experimental results suggested that troponin T might be the promising target protein of the aptamer AraHH001. Additionally, we carried out an EMSA analysis of AraHH001 with cTnT to determine binding capacity. The binding results for a gel shift assay of the aptamer with cTnT (Fig.2C) also provided strong evidence that AraHH001 is the target protein troponin T.

**Figure 3:**
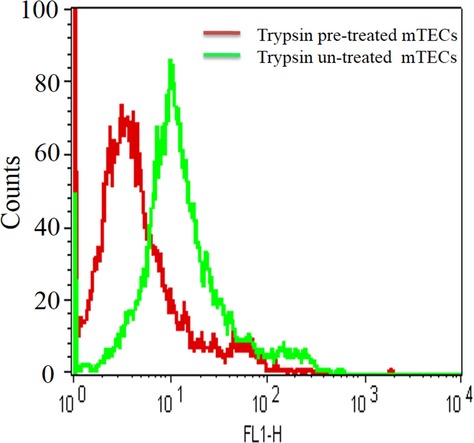
A flow cytometry assay of troponin T expression on trypsin pretreated mTECs and trypsin untreated mTECs. In flow cytometry binding assay, red and green curve represents the binding of the anti-troponin T antibody with mTECs pretreated with or without 0.1× trypsin, respectively.

**Figure 4:**
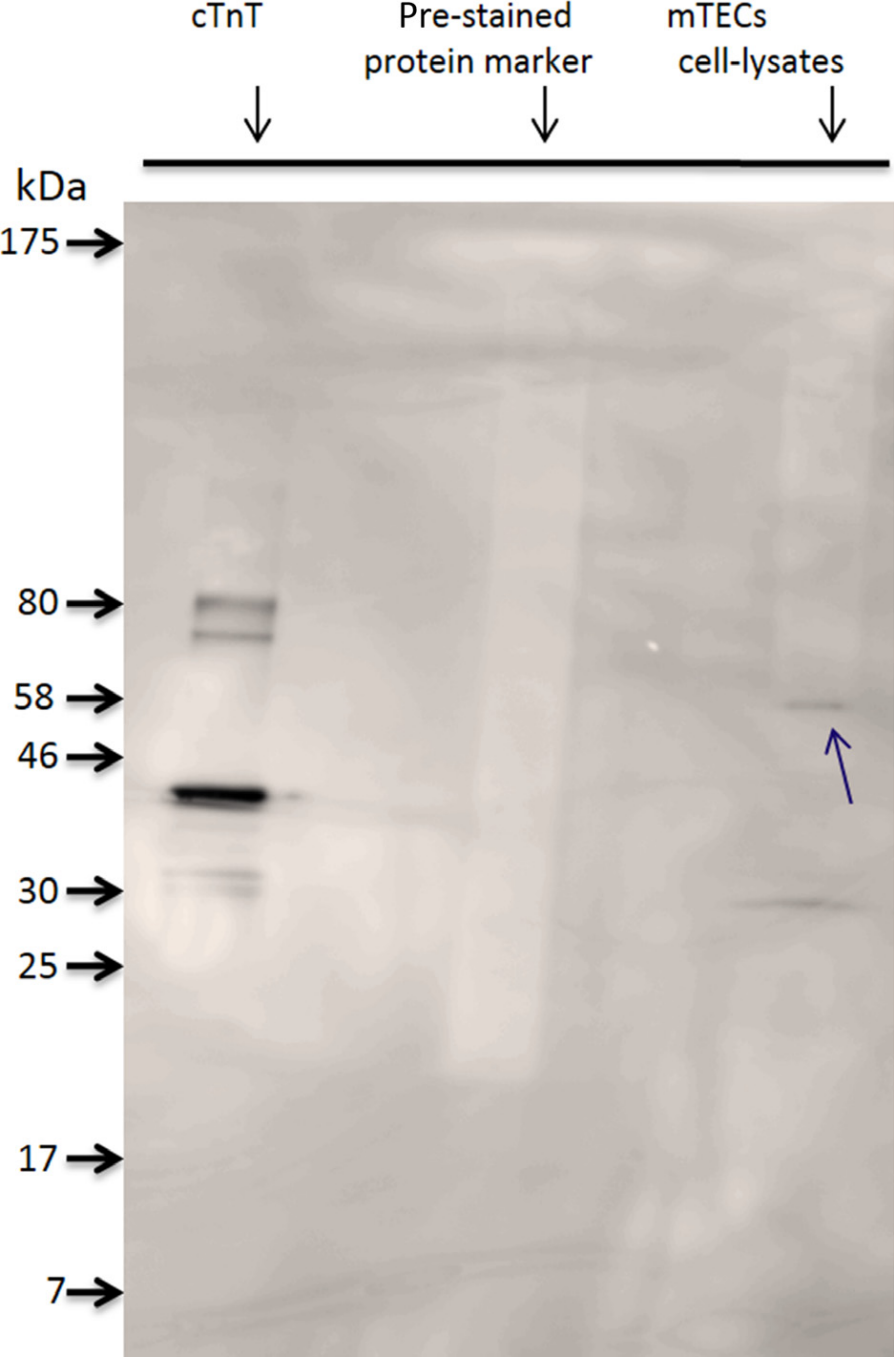
Western blot analysis of detecting troponin T protein from mTECs cellular extracts contains membrane proteins. In WB analysis, cardiac TnT in first lane recognized by anticardiac troponin T (cTnT) antibody. In the second and third lane, nonrecognized prestained protein marker and recognized troponin T from mTECs cellular lysates by anti-cTnT antibody.

We then confirmed the membrane-bound protein to be troponin T by trypsin pretreated cells, followed by the use of flow cytometry (Fig.3). From the above series of experimental results, we conclude that troponin T is expressed on mTECs surfaces. Since the molecular weight of identifying aptamer targets protein troponin T found 33 kDa, and it appeared near 60 kDa in SDS gel pictures (Fig.1B), we also performed WB experiment to ensure that troponin T antibody could recognize the troponin T protein directly from mTECs cellular extracts (Fig.4). WB experiments also recognized troponin T near 60 kDa as a dime, (Fig.4) the same as was observed in the SDS-PAGE picture (Fig.1B). It can also therefore be concluded that the AraHH001 target protein troponin T with an approximate molecular weight of 33 KD, is present on mTECs and that it is the target protein of the DNA aptamer AraHH001.

**Figure 5:**
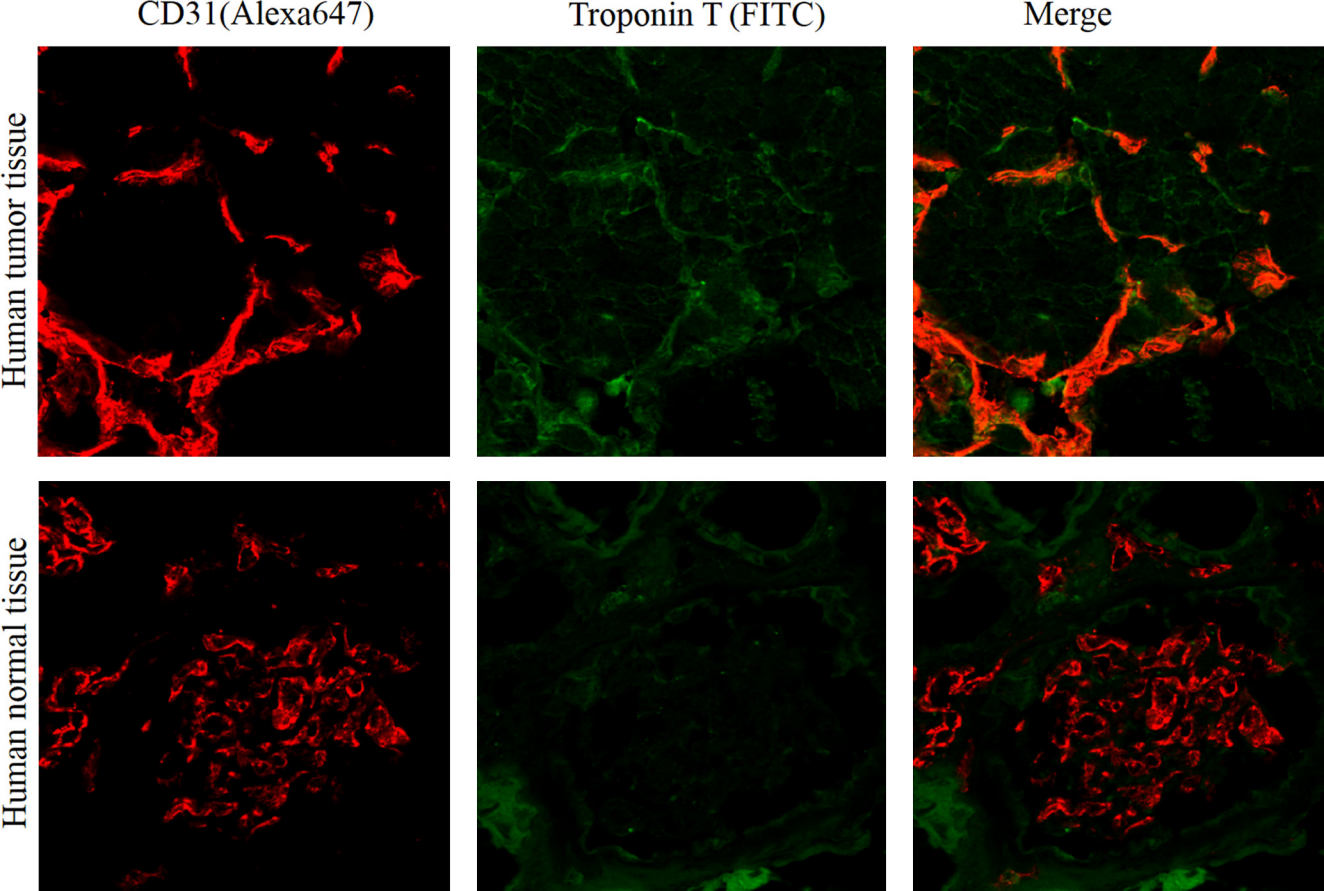
Expression of troponin T on human tumor blood vessel. In immunostaining staining experiment, cardiac troponin T as green fluorescence signal expressed in human tumor blood vessels. Positive control, CD31 with red signal was shown as red. Both green and red signal showing as merge.

Furthermore, we observed the expression of tropnin T in human renal carcinoma patient tissue sample in immunostaining experiment (Fig.5). Green fluorescence signal of tropnin T antibody was merged with red CD31 used as positive control. We have already reported that our novel DNA aptamer AraHH001can bind not only mTECs but also human TECs 44. Our findings about the troponin T expression on human tissue sample is very consistent over other experiments, and exhibit strong evidence that Trponin T, a novel biomarker of TECs.

The use of aptamers as protein-affinity reagents has a considerable advantage over the use of antibodies, due to its low cost, availability, and ease of chemical modification. Until now, the early diagnosis of tumors that are associated with various types of cancers has been difficult, due to the lack of appropriate diagnostic reagents and, or, specific markers or, specific targeting drug delivery systems 14,19,54. Our goal in this study was to identify a new marker from mTECs that can be used to reliably predict developing tumor metastasis. Aptamer-assisted biomarker identification is a strategy that offers opportunities for discovering the functions of many unknown surface proteins that are present in TECs. We considered that the identification of troponin T represents a good example of this. Recently other researcher groups have also reported the identification of aptamers, and have been able to recognize aptamer membrane-bound proteins from live cells, such as the PTK7 protein targeting the sgc8 aptamer 31,32.

In this study, we exploited a situation in which, if the aptamer affinity is high in the nmol/L range, a biotin-tagged aptamer is required to purify the target protein without causing any complications in the MALDI-TOF analysis. For validation of the identified protein, we showed that measurement of mRNA levels by qRT-PCR, gel shift assays, measurement of the protein expression of an antibody by flow cytometry, and WB experiment are useful tools. We believe that this versatile strategy of purifying a membrane-bound protein by a high-affinity ligand aptamer, and the associated validation techniques will stimulate other researchers to purify more differentially expressed surface markers of healthy cells and unhealthy cells under different patho-physiological conditions.

Generally troponin T is well-recognized as a cardiac marker for cardiac injury 55. Although cTnT is a highly sensitive marker, it is not absolutely cardiac specific. Elevated levels have been demonstrated in about 30% of all patients with end stage renal failure, but the exact reason for this still remains unknown 56. To date, there is not a single report regarding the presence of troponin T on mTECs. Although the exact reason, and its mechanistic role on mTECs is still unknown, there are some factors that are known to stimulate the release of troponins, including oxidative stress, inflammatory cytokines, etc. 57. These cytokines and chemo-attractants are not only secreted by immune regulatory cells but also by tumor cells, tumor-associated macrophages, and stromal cells. Together, these factors establish a microenvironment that promotes tumor progression by stimulating processes such as angiogenesis, invasion, and metastasis. Importantly, cytokines that are secreted by tumor cells also recruit tumor infiltrating leukocytes. These leukocytes can produce either pro- or antitumorigenic/angiogenic cytokines, which also play a role in determining how tumor growth is affected 50–58. In myocardial infarction, troponin levels have been reported to stimulate the production of the VEGF receptor family 58. Our hypothesis is the possibility of interrelationships between the VEGF receptor family and troponins, and, or troponins have a VEGF-like function on mTECs cannot be excluded. The most advanced antiangiogenesis agents in the clinic are the anti-VEGF antibody Avastin and a VEGF165 aptamer, Macugen 59–62. We hypothesize that a novel protein troponin T that targets AraHH001 has great potential for determining the exact reason and its mechanistic role on mTECs as well as in antiangiogenesis therapy. It might exert a dramatic change in the pace of cancer research and could have a dramatic impact in diagnosis on cancer patient care in the future.

## Supporting Information

**Figure S1.** Schematic representation of the affinity purification of an AraHH001-targeted protein. (1) Lyses the cells by lyses buffer with protease inhibitors to collect protein solution. (2) Incubation of aptamer with protein solution without linker or with linker. (3) Collection of bound aptamer–protein complex or aptamer-link–protein complex. (4) Incubate aptamer–protein complex or aptamer-link–protein complex with MB for 20 min on ice. (5) Elute aptamer-bound protein by heating 95°C for 5 min. (6) Run on SDS-PAGE, stained with silver for analysis. **Figure S2.** Identification of a DNA aptamer AraHH001-targeted protein via PMF analysis (A) MALDI-TOF mass spectrum of purified target protein troponin T with detail information of 16 fragments matched with troponin T (Table S1). (B) Mascot score histogram. Troponin T matched peptides shown in bold red and the total sequence coverage was 47%, showing protein scores greater than 72% are significant. **Figure S3.** Similarity search between slow skeletal muscle troponin T and cardiac troponin T. Identified troponin T was shown 70% similarity with cardiac troponin T. **Figure S4.** Similarity search between slow skeletal muscle troponin T and cardiac troponin T. Identified troponin T was shown 70% similarity with cardiac troponin T. **Table S1.** Troponin T fragments, identified by MALDI-TOF mass analysis.
