# Supplementary material for: Identification and expression of troponin T, a new marker on the surface of cultured tumor endothelial cells by aptamer ligand
Source: Cancer Med. 2014 May 9;3(4):825–34. doi: 10.1002/cam4.260 (PMC4303150; doi:10.1002/cam4.260)
Supplement: Supplementary file 1 [file cam40003-0825-sd1.docx]

**Supplemental Online Materials**

**Supplemental Figure 1**


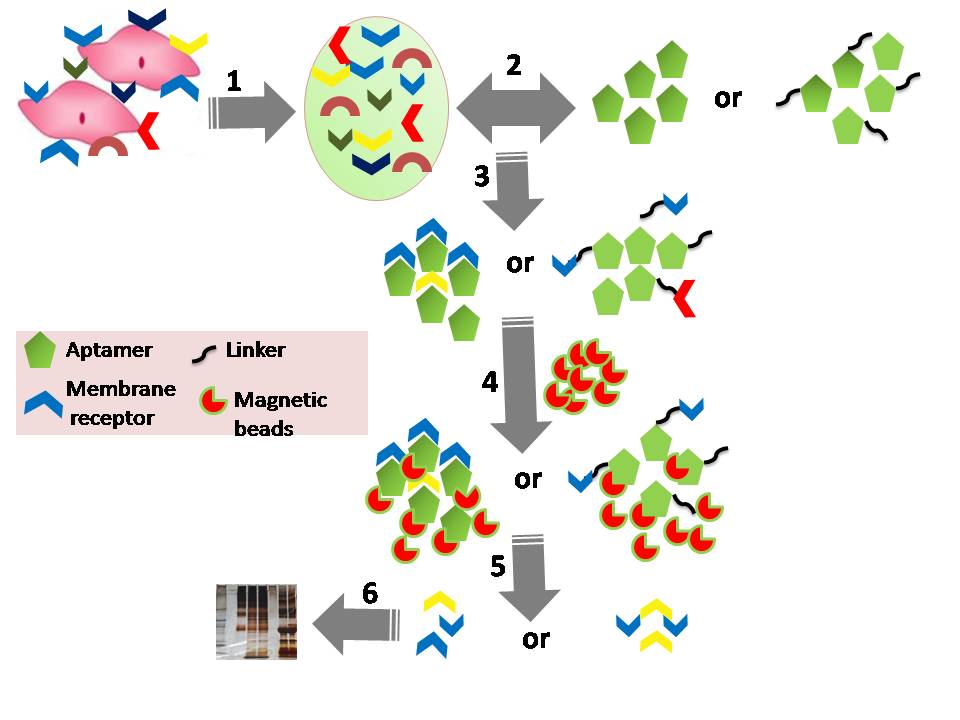


**Supplemental Figure 1: Schematic representation of the affinity purification of an AraHH001 targeted protein.** **1.** Lyses the cells by lyses buffer with protease inhibitors to collect protein solution. **2.** Incubation of aptamer with protein solution without linker or with linker 3. Collection of bound aptamer-protein complex or aptamer-link-protein complex **4.** Incubate aptamer-protein complex or aptamer-link-proten complex with MB for 20 mins on ice. **5.** Elute Aptamer bound protein by heating 95^0^ C for 5minutes. **6.** Run on SDS PAGE, stained with silver for analysis.

Table 1: Troponin T fragments, Identified by MALDI-TOF mass analysis.

| **Pptide** | **Observed**  **Mass (Da) ^a^** | **Expected**  **Mass (Da) ^b^** | **Calculated**  **Mass (Da) ^c^** | **Miss** | **Position^d^**  **(Start-End)** |
| --- | --- | --- | --- | --- | --- |
| 1 | 2165.21 | 2164.20 | 2164.23 | 0 | 35-53 |
| 2 | 1697.78 | 1696.78 | 1697.77 | 1 | 54-67 |
| 3 | 1016.49 | 1015.48 | 1015.47 | 0 | 60-67 |
| 4 | 1144.57 | 1143.56 | 1143.56 | 1 | 60-68 |
| 5 | 2357.20 | 2356.19 | 2356.20 | 1 | 70-88 |
| 6 | 2373.25 | 2372.25 | 2373.20 | 1 | 70-88 |
| 7 | 1668.89 | 1667.89 | 1668.03 | 0 | 73-88 |
| 8 | 1592.76 | 1591.75 | 1591.81 | 0 | 152-166 |
| 9 | 1608.78 | 1607.77 | 1607.81 | 0 | 152-166 |
| 10 | 1819.86 | 1818.85 | 1818.92 | 1 | 189-203 |
| 11 | 1835.90 | 1834.89 | 1834.89 | 1 | 189-203 |
| 12 | 2590.31 | 2589.30 | 2589.24 | 1 | 206-226 |
| 13 | 2606.33 | 2605.32 | 2605.23 | 1 | 206-226 |
| 14 | 1567.77 | 1566.77 | 1566.81 | 1 | 229-240 |
| 15 | 1183.60 | 1182.59 | 1182.60 | 0 | 232-240 |
| 16 | 986.55 | 985.54 | 985.54 | 1 | 241-248 |

**^a^** Molecular mass observed after MALDI-TOF mass spectrometry analysis

**^b^** Expected relative molecular mass of the matched peptide

**^c^** Calculated relative molecular mass of the matched peptide

**^d^** Position of the peptide inside the Troponin T sequence

**Supplemental Figure 2 A**


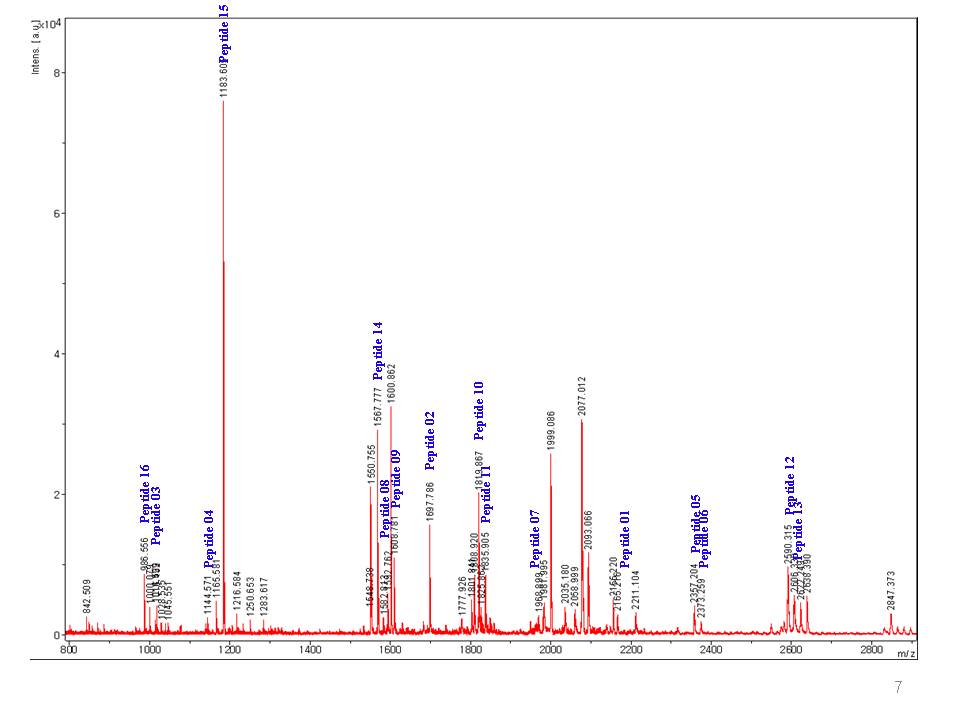


Mass (m/z)

Intensity

**Supplemental Figure 2 B:**

**
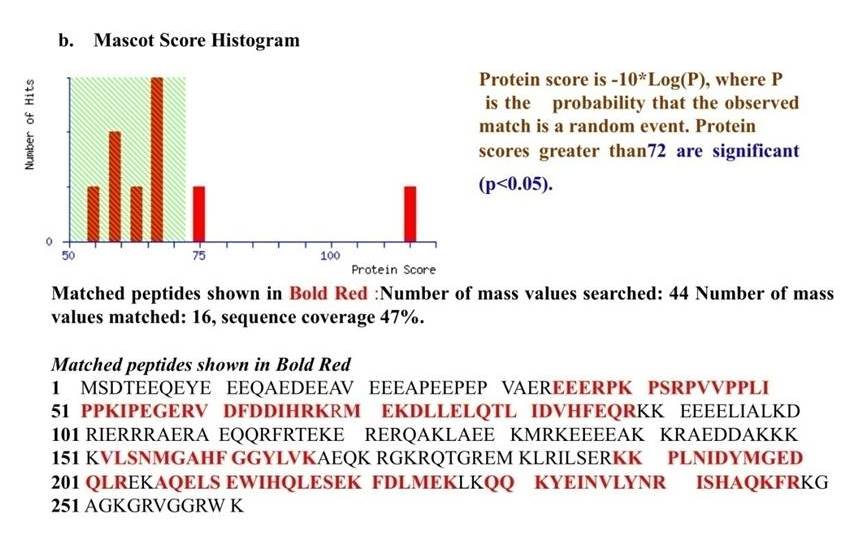
**

**Supplemental Figure 2:** **Identification of a DNA aptamer AraHH001 targeted protein via PMF analysis (**A). MALDI-TOF mass spectrum of purified target protein Troponin T with detail information of 16 fragments matched with Troponin T (Listed in Table 1).

(B**)** Mascot Score Histogram. Troponin T Matched peptides shown in bold red and the total sequence coverage was 47%, showing protein scores greater than 72% are significant.

**Supplemental Figure 3:
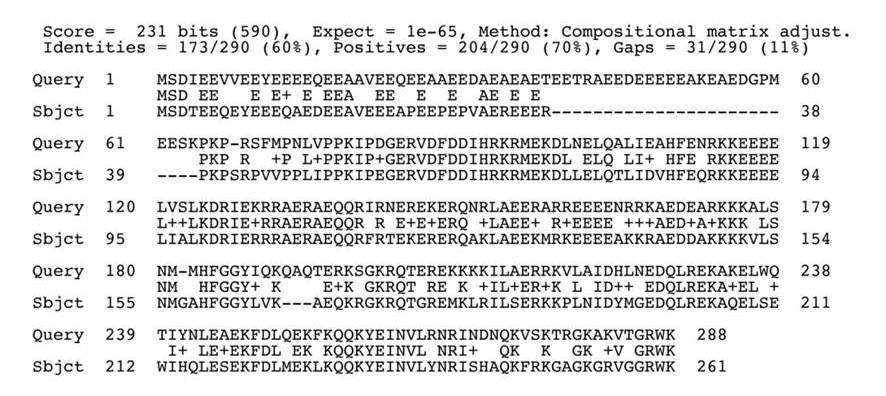
**

**Supplemental Figure 3:** Similarity search between slow skeletal muscle Troponin T and cardiac Troponin T. Identified Troponin T was shown 70% similarity with cardiac Troponin T.
